# How TikTok Influencers Disclose Food and Beverage Brand Partnerships: Descriptive Study

**DOI:** 10.2196/60891

**Published:** 2025-02-28

**Authors:** Roxanne Dupuis, Aviva A Musicus, Brittany Edghill, Emma Keteku, Marie A Bragg

**Affiliations:** 1 Department of Population Health NYU Grossman School of Medicine New York, NY United States; 2 NYU Food Environment and Policy Research Coalition New York, NY United States; 3 Department of Nutrition Harvard T.H. Chan School of Public Health Boston, MA United States; 4 Center for Science in the Public Interest Washington, DC United States; 5 Office of Science and Research NYU Grossman School of Medicine New York, NY United States; 6 New York Presbyterian Hospital-Weill Cornell New York, NY United States; 7 Marketing Department Stern School of Business New York University New York, NY United States

**Keywords:** social media, social media marketing, social media influencer, food and beverage marketing, adolescent health

## Abstract

**Background:**

Food and beverage marketing is an important influence on the health and diets of adolescents. Food and beverage companies spend billions of dollars annually on advertisements to promote their products and are increasingly focusing on social media influencers. Influencer product endorsements blur the line between entertainment and marketing.

**Objective:**

This study aimed to quantify how often TikTok influencers promote products from food and beverage brands and document the range of ways they disclose brand relationships in their content.

**Methods:**

We collected up to 100 videos posted on or before July 1, 2022, from each of the top 100 influencers on TikTok in the United States and recorded information about the influencer (eg, number of followers) and video (eg, number of views and likes). For each video that contained food or beverage products, we identified the main product featured. A team of research assistants then coded each video for how the product was featured (ie, in the video, audio, or caption) and, for branded products, whether the video was accompanied by any disclosures of brand relationships. Average pairwise percentage agreement among coders was 92%, and average pairwise Cohen κ was 0.82.

**Results:**

Among the 8871 videos from 97 influencers that made up the final analytical sample, we identified 1360 videos (15.3%) that featured at least one food or beverage product. These 1360 videos were viewed >9 million times and received >1 million likes each. Nearly half (n=648, 47.6%) of the videos featured a branded product. Most videos featuring a branded product did not contain a brand relationship disclosure (n=449, 69.3%). Among videos that disclosed a brand relationship, influencers used 10 different types of disclosures. Tagging a brand in the video’s caption was the most common disclosure method (n=182, 28.1%). Six types of caption hashtags were used to disclose brand relationships, including #[brandname] (n=63, 9.7%) and #ad (n=30, 4.6%). Only 1 video (0.2%) made use of TikTok’s official disclosure label and only 1 video (0.2%) verbally mentioned a contractual agreement with a brand.

**Conclusions:**

Among the food and beverage videos with disclosures we identified, the most frequently used mechanism—tagging the brand—did not clearly differentiate between sponsored content and the influencer trying to attract a brand or followers who may like that brand. Social media users, particularly adolescents, need clearer, more robust disclosures from influencers to protect against the undue influence of food marketing. These findings may also inform calls for the Children’s Food and Beverage Advertising Initiative—the largest self-regulatory pledge to reduce unhealthy food marketing—to include older adolescents, who are heavily targeted by food and beverage companies on social media.

## Introduction

Food and beverage companies spend billions of dollars annually on advertisements to promote their products [1]. Food marketing (including beverages) is an important influence on the health and diets of children and adolescents. Advertisements affect brand preferences and eating behaviors [2]. Experimental evidence shows that children and adolescents consume significantly more calories when viewing television advertisements featuring unhealthy foods compared to nonfood products [3-5]. In 2006, the Institute of Medicine recognized food marketing toward children as “out of balance” with healthy eating, putting children and adolescents’ health at risk [1].

Most food advertisement studies have focused on television [1,3-6], but adolescents are shifting toward digital entertainment and social media [7]. Half (51%) of adolescents in the US spend an average of 4.8 hours daily on social media [8]. One popular social media platform among adolescents is the short video–sharing app TikTok. The social media company’s internal documents indicate that, in 2020, one-third of their 49 million US daily users were younger than 14 years [9]. Since then, the app’s popularity has grown exponentially, with 150 million users in the United States, excluding children under the age of 13 years [10]. TikTok’s growing popularity makes it poised to overtake YouTube as the most-used app among adolescents [11], but to date there is little research on the effect of food advertisements on this platform [12-14].

Following this shift in media consumption by adolescents, food and beverage companies are increasingly focusing on social media marketing [15]. A study in Australia found that adolescents were exposed to roughly 17 food advertisements for every hour they spent on the internet or on social media and video-sharing apps on their phones [16]. One food company advertising strategy is to pay “influencers,” who are “regular, everyday people” who become online celebrities with large followings, with posts that generate billions of views, to promote products either discreetly or overtly on their social media accounts [14,17]. Adolescents are especially susceptible to social media advertisements from influencers [18] because they are likely to evade adolescents’ media literacy skills. The processing of commercialized media content model [19] purports that when children recognize what they are seeing as a commercial, they are able to use their defenses (eg, skepticism, recognition of persuasive intent) to reduce an advertisement’s influence. But influencers blur the boundary between entertainment and marketing, which may reduce adolescents’ ability to recognize and defend against the persuasive intent associated with their messaging. Because social media influencers are seen as regular, everyday people, their product endorsements may be experienced as credible recommendations rather than commercial advertisements [20].

The Federal Trade Commission (FTC), the government agency that regulates marketing on social media platforms in the United States, requires influencers to disclose sponsored posts, but these disclosures may not operate as intended. The FTC permits very subtle disclosures, such as adding the words “#ad” or “#sponsored” at the bottom of posts [21], so viewers may not even notice them among the other text surrounding the image.

Little is known about how TikTok influencers disclose food brand partnerships. Our goal in this study was to quantify how often TikTok influencers promote products from food brands and document the range of ways they disclose brand relationships in their content.

## Methods

### Data Collection

We collected up to 100 videos posted on or before July 1, 2022, from each of the top 100 influencers on TikTok in the United States. Each video was collected by capturing the full video and captions on a research assistant’s phone. Research assistants noted the date the video was posted and the numbers of views, likes, comments, and “saves” or bookmarks. They also collected profile information, such as whether the influencer was “verified” with a blue checkmark and the total number of likes and followers.

### Ethical Considerations

This study was determined to be non–human subjects research and used preexisting data that included publicly available information and therefore does not require institutional review board review per Federal Regulations for the Protection of Human Research Subjects (45 CFR 46.104(d)(4)) [22]. TikTok allows users to select whether their accounts are public or private. All collected data (ie, videos) were publicly available to anyone on TikTok. No user (ie, follower) data were collected. Only deidentified and aggregated data are presented.

### Content Analysis

Using a 3-step approach, we first identified whether a food product was featured. Second, we identified the main product, based on whether it was (1) the only featured food product; (2) a sponsored product; or (3) the most prominent or dominant product (eg, the first to appear or to grab the viewer’s eye). For each main product, the research team then noted whether it was branded (eg, Coke bottle vs banana). Third, a team of research assistants viewed each food product video to capture information about how the product was featured (ie, in the video, caption, or audio) and, for any branded product, where the brand or logo was featured and whether the video was accompanied by any brand disclosures.

### Statistical Analysis

Using ReCal3 [23], we calculated interrater reliability among coders in the third step using a small sample of “practice” videos (n=6) that reflected the range of disclosures used [24]. Average pairwise percentage agreement was 92%, and average pairwise Cohen κ was 0.82. We deemed these to be acceptable given the descriptive and exploratory nature of this work. We report descriptive statistics.

## Results

From the initial potential pool of 10,000 videos across 100 influencers, we had a final analytical sample of 8871 videos from 97 influencers (Multimedia Appendix 1, Figure S1). We identified 1360 videos (15.3%) that featured at least one food product. These 1360 videos originated from 89 unique influencers (median 9, IQR 4-21 videos per influencer), each with >17 million followers and >526 million profile likes. The videos themselves were viewed >9 million times and received >1 million likes (Table 1).

**Table 1:** Follower engagement with videos featuring food or beverage products, overall and for branded products only.

Engagement	Featuring all food or beverage products (n=1360), mean (SD)	Featuring branded food or beverage products (n=648), mean (SD)
Views	9,249,401 (14,963,961)	8,924,298 (15,584,292)
Likes	1,062,903 (1,777,784)	1,099,153 (1,826,587)
Comments	7524 (14,437)	7189 (13,530)
Bookmarks or “saves”	30,144 (59,915)	33,542 (67,666)

Nearly half (n=648, 47.6%) of the videos with food products featured a branded product. Product brands or logos were most often featured in the video itself (n=606, 93.5%), followed by the video caption (n=224, 34.6%), and/or the accompanying audio (n=126, 19.4%).

Most videos featuring a branded food or beverage product did not contain a brand relationship disclosure (n=449, 69.3%). Among videos that disclosed a brand relationship, influencers used 10 different types of disclosures (Table 2). Tagging a brand in the video’s captions was the most common disclosure method (n=182, 28.1%). Six different types of caption hashtags were used to disclose brand relationships, including #[brandname] (n=63, 9.7%) and #ad (n=30, 4.6%).

**Table 2:** The 10 different types of brand relationship disclosures used in videos featuring branded food and beverage products^a^ (n=648).

Types of disclosure			Disclosures, n (%)
**No disclosure**			449 (69.3)
**Caption hashtags (#)**			
	#[brandname]	63 (9.7)	
	#ad	30 (4.6)	
	#[brand]partner	22 (3.4)	
	#[brand]ambassador	3 (0.5)	
	#sponsored or #sponsorship	1 (0.2)	
	Other hashtag (eg, brand slogan)	39 (6.0)	
**Brand tags (@)**			
	Brand tagged in video	4 (0.6)	
	Brand tagged in caption	182 (28.1)	
**Official TikTok disclosure label**			1 (0.2)
**Contractual agreement verbally disclosed**			1 (0.2)

^a^Not all disclosures represent actual brand relationships; disclosures are not mutually exclusive.

## Discussion

### Principal Results

Nearly half of the TikTok posts by top influencers that included food and beverage products featured a branded product. The majority (69.3%) of these posts, however, did not include any brand relationship disclosures, suggesting either that no relationship existed or that they purposely or accidentally omitted its disclosure. By comparison, a recent study of food-related Instagram posts by celebrities (athletes, actors, and musicians) found that less than 5% of the identified posts that were sponsored by a food company were identified by hashtags (eg, “#ad”) or related terms (eg, “paid advertisement”) below the post [25].

Among the food videos with disclosures we identified, the most frequently used mechanism—tagging the brand—did not clearly differentiate between sponsored content and the influencer trying to attract a brand or followers who may like that brand. These findings have implications for how social media marketing should be regulated. The ambiguity around brand relationship disclosures makes it difficult for social media users, particularly adolescents, to make informed decisions about the products they see [18]. This concern has been recognized by the FTC in the most recent iteration (July 2023) of its endorsement guides [26], which emphasize that marketing practices geared toward adults may not be appropriate for children. In fact, bigger and clearer disclosures would likely work better among children and adolescents. A qualitative study among participants aged 12-16 years reported that they did not mind if influencers were paid, but they did not like seeing explicit disclosures that were “...too pushy, like, this is advertising, because then you will keep this in mind all the time” [27].

### Limitations

Given the dearth of information on influencer marketing, our study set out to systematically collect and record information on how social media influencers disclose relationships with food brands. However, our study has limitations, including (1) the use of a convenience sample of influencers and posts, (2) the focus on only the most prominent food product in each video, (3) the inability to infer intentionality in whether a product was meant to be featured in a video (eg, when the only food product featured was in the background), and (4) the inability to distinguish whether the presence or lack of a disclosure implied the presence or lack of a brand relationship.

### Conclusions

Social media users, particularly adolescents, need clearer, more robust disclosures from influencers to protect against the undue influence of food marketing. These findings may inform calls for the Children’s Food and Beverage Advertising Initiative—the largest self-regulatory pledge designed to reduce unhealthy food marketing—to extend its protections to older adolescents, who are heavily targeted by food companies [28,29].

## Appendix

Multimedia Appendix 1 Data collection and cleaning flow chart.

## Abbreviations

FTC: Federal Trade Commission

## Data Availability

The datasets generated or analyzed during this study are available from the corresponding author on reasonable request.
