# Supplementary figures and images for: How TikTok Influencers Disclose Food and Beverage Brand Partnerships: Descriptive Study
Source: J Med Internet Res. 2025 Feb 28;27:e60891. doi: 10.2196/60891 (PMC11909480; doi:10.2196/60891)

**Multimedia Appendix 1.** Data collection and cleaning flow chart.

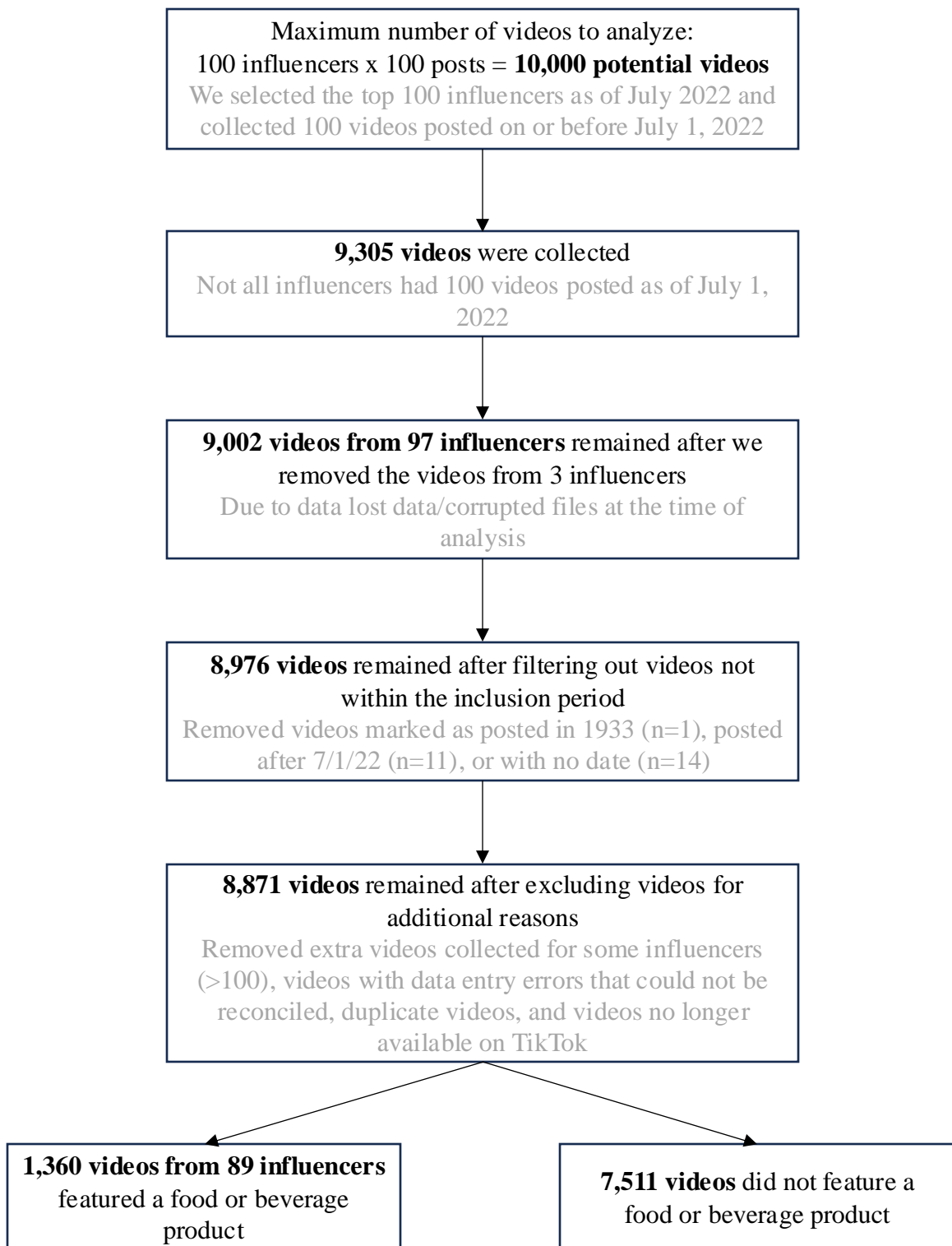

Supplement: Multimedia Appendix 1 [file jmir_v27i1e60891_app1.pdf]
